# Prevalence and predictors of detrusor underactivity and bladder outlet obstruction in women with lower urinary tract symptoms

**DOI:** 10.1038/s41598-024-76242-y

**Published:** 2024-10-24

**Authors:** Chin-Jui Wu, Sheng-Mou Hsiao, Pei-Chi Wu, Ting-Cheng Chang, Chi-Hau Chen, Bor-Ching Sheu, Ho-Hsiung Lin

**Affiliations:** 1 Department of Obstetrics and Gynecology, National Taiwan University Hospital Hsin-Chu Branch, Hsin-Chu City, 300195 Taiwan; 2 Department of Obstetrics and Gynecology, National Taiwan University College of Medicine and Hospital, No. 8, Zhongshan S. Rd., Zhongzheng Dist., Taipei City, 100225 Taiwan; 3 Department of Obstetrics and Gynecology, Far Eastern Memorial Hospital, No. 21, Sec. 2, Nanya S. Rd., Banqiao Dist., New Taipei City, 220216 Taiwan; 4 Graduate School of Biotechnology and Bioengineering, Yuan Ze University, No. 135, Yuandong Rd., Zhongli Dist., Taoyuan City, 320315 Taiwan

**Keywords:** Detrusor underactive, Bladder outlet obstruction, Lower urinary tract symptoms, Urodynamics, Medical research, Urology

## Abstract

This study aims to present age-stratified prevalence of women with lower urinary tract symptoms (LUTS) but without cystocele and predict detrusor underactivity (DU) or bladder outlet obstruction (BOO). Between 2005 and 2020, we reviewed women who visited the medical center with LUTS but without cystocele. Positive voiding dysfunction (VD) symptoms were defined as any one or more of the positive descriptions of weak urinary stream, intermittency, strain to urination, and sensation of not emptying. A total of 1,886 women were included in this study. 189 (10.0%) women were diagnosed with DU, and 77 (4.1%) women had BOO. Multivariate logistic regression analysis found that voided volume and VD symptoms were independent predictors for BOO. ROC curve analyses could predict BOO by voided volume ≤ 220 mL derived from uroflowmetry and the presence of VD symptoms with an area under a curve of 0.83. Age and voided volume could predict DU with an area under a curve of 0.82. We found a higher percentage of BOO in women with positive VD symptoms. A non-invasive uroflowmetry with voided volume (≤ 220 mL) and the presence of VD symptoms can predict BOO. DU could be predicted by age and voided volume.

## Introduction

Female voiding dysfunction (VD) needs a clear and objective definition. The prevalence varies widely from 2.7 to 23% according to various criteria^1^. Both clinical storage and voiding symptoms correlate with VD, including urgency, frequency, weak stream, and straining. The International Continence Society (ICS) defined VD as “abnormally slow and/or incomplete micturition, based on abnormally slow urine flow rates and/or abnormally high post-void residuals, ideally on repeated measurement to confirm abnormality. Pressure-flow studies can be required to determine the cause of the voiding dysfunction.”^2^ Nowadays, urodynamic study (UDS) is the gold standard for clarifying female VD and categorized functional detrusor underactivity (DU), and bladder outlet obstruction (BOO)^3^.

DU has represented a low-pressure-low-flow pattern in UDS with poor voiding efficacy and substantial post-void residual urine. BOO can be anatomical or functional and is characterized by a high-pressure-low-flow pattern in pressure-flow studies^4,5^. Because of the high complexity of urinary symptoms to correlate with symptoms, Dr. Bates et al. wrote that the bladder is an unreliable witness^6^. Thus, most women presenting lower urinary tract symptoms (LUTS) require an invasive pressure-flow study, part of the UDS, to differentiate DU or BOO. A non-invasive prediction model including symptoms of DU and BOO is still lacking.

Our study aimed to calculate the age-stratified prevalence of DU and BOO in women with LUTS. We divided the LUTS into the presence or absence of clinical VD symptoms. We utilized four symptoms including weak urinary stream, intermittency, strain to urination, and sensation of not emptying. Patients fulfilled one of the four symptoms were defined positive clinical VD symptom. We aimed to calculate an office-based prediction model to facilitate clinicians’ identification of these patients.

## Results

**Fig. 1:**
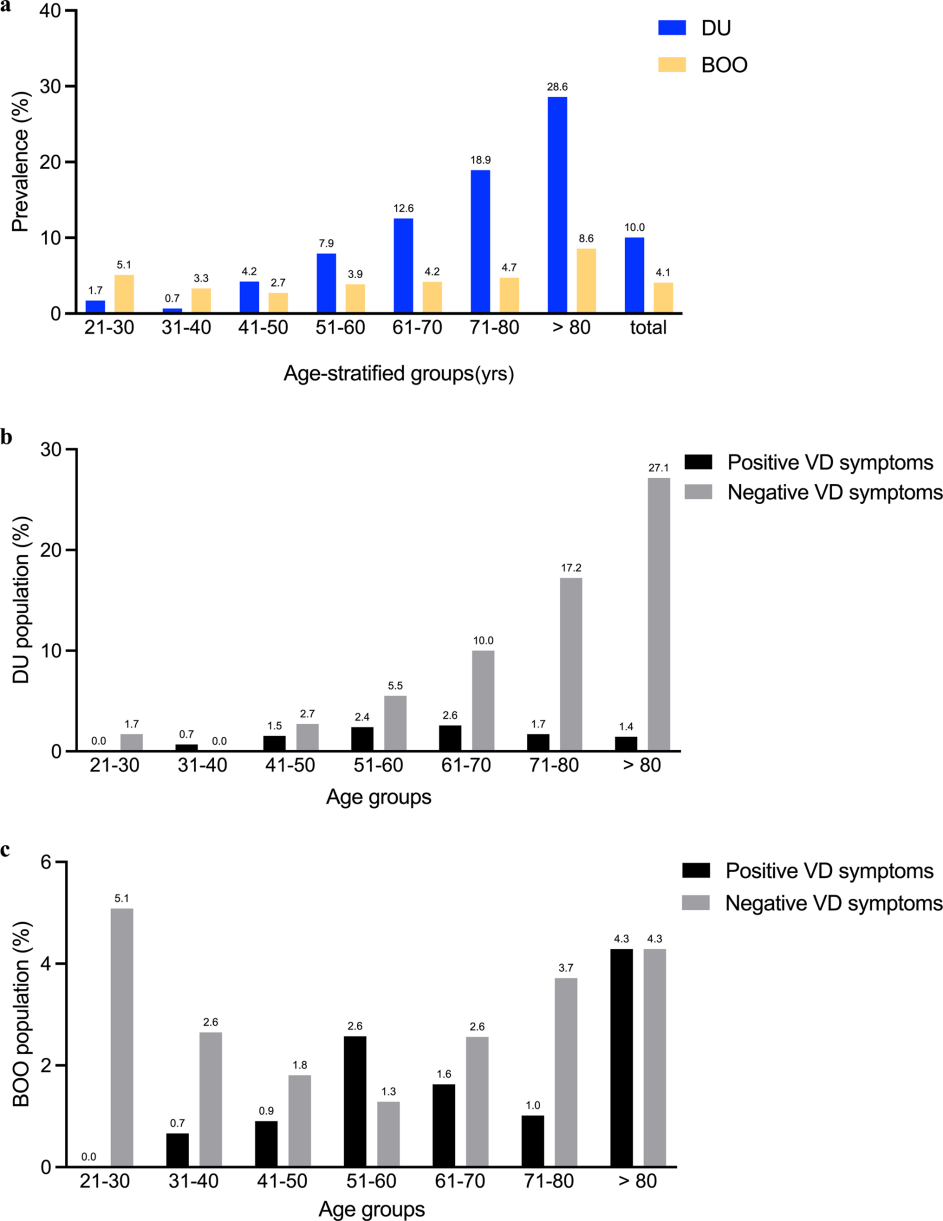
The prevalence of detrusor underactivity (DU) and bladder outlet obstruction (BOO) is divided by age per 10 years. **a** The age-stratified prevalence of DU and BOO diagnosis in all 1,886 women. **b** The age-stratified DU population in clinical positive voiding dysfunction (VD) and negative VD symptoms. **c** The age-stratified BOO population in clinical positive voiding dysfunction (VD) and negative VD symptoms.

The mean age of the total enrolled 1,886 women was 57.7 years old. For those diagnosed with DU or BOO women, the mean age was 66.2 and 59.5 years old, respectively. The total prevalence of women who presented with LUTS was 10% (189/1886) for DU and 4.1% (77/1886) for BOO. The age-adjusted prevalence rates of DU and BOO are shown in Fig. 1a. The DU patients showed an increasing trend with age, while the BOO patients kept a similar proportion throughout all age stratifications. The proportion did not increase with age in DU patients with clinical VD symptoms. In comparison, DU patients without clinical VD symptoms did increase their prevalence with age (Fig. 1b, *p* = 0.031). In the BOO groups, women with and without symptoms of VD did not show such trend of DU (Fig. 1c, *p* = 0.156).

In women with VD symptoms, 12.8% (36/282) were diagnosed with DU, and 11.3% (32/282) had BOO, according to the criteria^7^. In those without VD symptoms, 9.5% (153/1604) had DU, and 2.8% (45/1604) had BOO. A significant difference can be found in the diagnosis of BOO (*p* < 0.001). Women with clinical symptoms of VD had a higher chance of being diagnosed with BOO than those without symptoms (Table 1). On the contrary, DU is relatively hard to determine from the clinical VD symptoms only.

**Table 1:** Prevalence of detrusor underactivity and bladder outlet obstruction in women with and without symptoms of voiding dysfunction (*n* = 1,886).

Variables	With symptoms of voiding dysfunction (*n* = 282)	Without symptoms of voiding dysfunction (*n* = 1604)	*P* ^a^
DU	36 (12.8)	153 (9.5)	0.096
BOO	32 (11.3)	45 (2.8)	< 0.001

Values are expressed by number (percentage). BOO = bladder outlet obstruction. DU = detrusor oversensitivity.

^a^Chi-square test.

To elucidate the factors predicting BOO, we performed univariate and multivariate analyses (Table 2). Primary demographic data and urodynamic study parameters were included. In the univariate analysis, the volume of strong desire (*p* = 0.001), maximal urethral closure pressure (MUCP) (*p* = 0.004), voided volume (*p* < 0.001), post-void residual urine (PVR) (*p* < 0.001), and symptoms of VD (*p* < 0.001) were significantly predicting factors associated with BOO, while the age, parity, pad test weight, functional urethral length, pressure-transmission ratio were not. Further multivariate analysis by stepwise backward logistic analysis identified that MUCP, voided volume, and symptoms of VD were the independent factors predicting BOO. The odds ratio is 3.64 for symptoms of VD, 0.0031 per mL for voided volume, and 1.009 for MUCP, respectively.

**Table 2:** Prediction of bladder outlet obstruction in women with lower urinary tract symptoms (*n* = 1,886).

Variables	Univariate analysis		Multivariable analysis	
	Odds ratio (95% CI)	P^a^	Odds ratio (95% CI)	P^b^
Age (years)	1.011 (0.994–1.028)	0.224	–	–
Parity	1.027 (0.891–1.184)	0.712	–	–
Pad weight (g)	0.998 (0.990–1.005)	0.554	–	–
Strong desire (mL)	0.608 (0.450–0.822)	0.001	–	–
MUCP (cmH_2_O)	1.009 (1.003–1.014)	0.004	1.009 (1.003–1.015)	0.005
FPL (cm)	1.070 (0.849–1.347)	0.567	–	–
PTR (%)	1.001 (0.996–1.006)	0.626	–	–
Voided volume (mL)	0.304 (0.226–0.409)	< 0.001	0.314 (0.234–0.422)	< 0.001
PVR (mL)	1.766 (1.395–2.234)	< 0.001	–	–
Symptoms of VD	4.434 (2.765–7.113)	< 0.001	3.637 (2.188–6.044)	< 0.001

Values are expressed by number (percentage).

CI = confidence interval; FPL = functional profile length; MUCP = maximum urethral closure pressure; PTR = pressure transmission ratio; PVR = post-void residual volume; VD = voiding dysfunction.

^a^Univariate logistic regression analysis.

^b^Multivariable Stepwise backward logistic regression analysis was performed using those variables at univariate analysis. Pseudo R^2^ = 0.20.

**Fig. 2:**
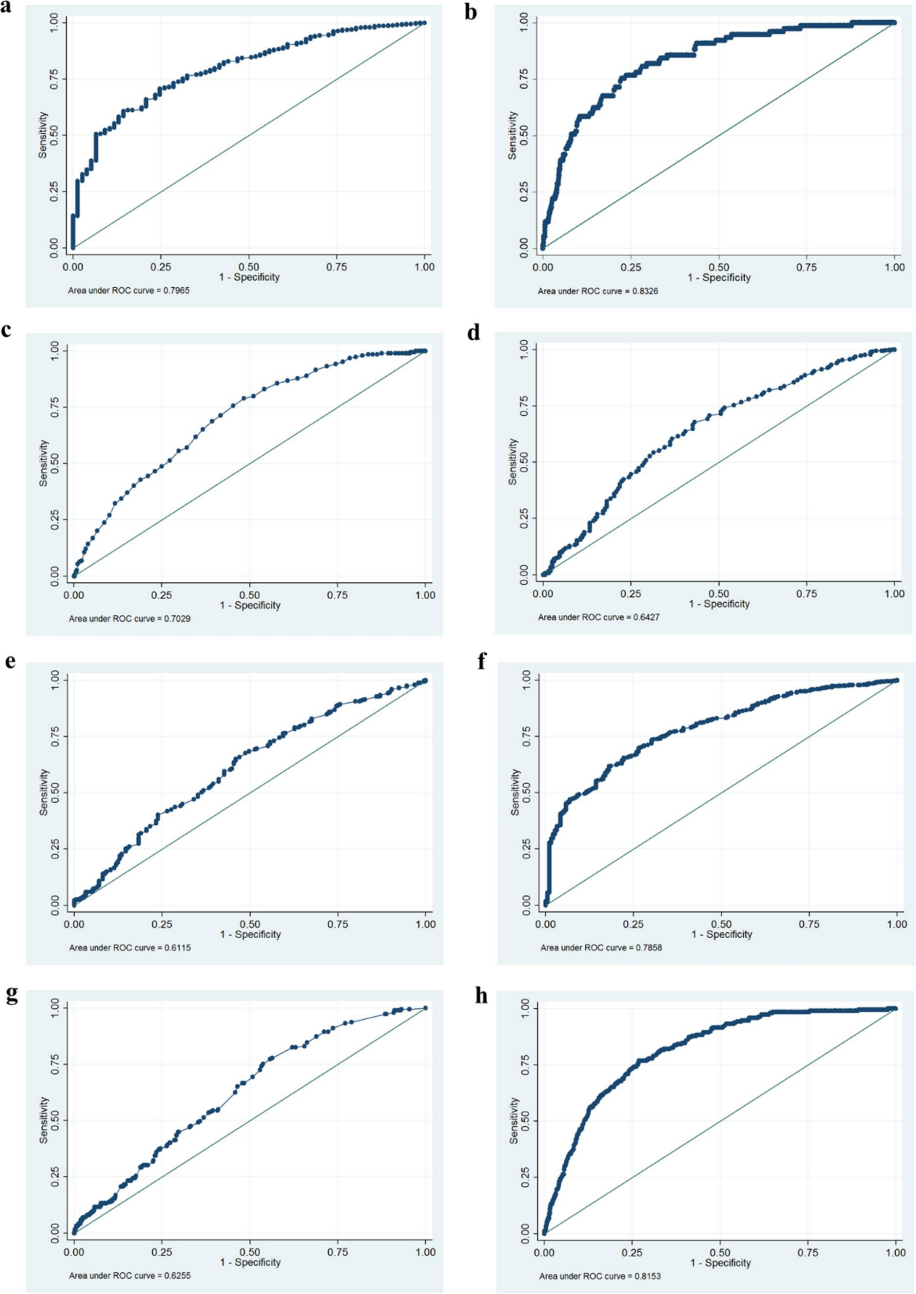
Clinical predictors of bladder outlet obstruction (BOO) and detrusor underactivity (DU) were calculated by receiver operating characteristic (ROC) curve analysis. **a** The ROC curve analysis using the single predictor voided volume ≤ 220 mL derived from uroflowmetry to predict bladder outlet obstruction. **b** Prediction of BOO by logit(p). Combined the predicted logit transformation of the probability of BOO, logit(p), for a voided volume (mL, a) and the presence of symptoms of VD (0 or 1, b) can be denoted by logit(p) = -1.3–1.1 x a + 0.014 x b, with a cut-off value of logit(p) ≥ -2.9. The area under the ROC curve is 0.83(95% CI = 0.79 to 0.88, sensitivity = 75%, specificity = 77%). **c** The ROC curve analysis using the single predictor age ≥ 60 years old to predict detrusor underactivity (95% CI 0.67 to 0.74, sensitivity = 71%, specificity = 58%). **d** The ROC curve analysis using the single predictor maximum urethral closure pressure ≤ 50 cmH2O to predict detrusor underactivity. (95% CI 0.60 to 0.69, sensitivity = 68%, specificity = 57%). **e** The ROC curve analysis using the single predictor functional profile length ≤ 2.5 cmH_2_O to predict detrusor underactivity (95% CI 0.57 to 0.66, sensitivity = 68%, specificity = 51%). **f** The ROC curve analysis using the single predictor voided volume ≤ 200 mL to predict detrusor underactivity (95% CI 0.76 to 0.82, sensitivity = 69%, specificity = 74%). **g** The ROC curve analysis using the single predictor post-void residual ≥ 30 mL to predict detrusor underactivity (95% CI 0.59 to 0.66, sensitivity = 83%, specificity = 38%). **h** Prediction of DU by logit(p). The predicted logit transformation of the probability of DU, logit(p), for age (year-old, a) and voided volume (mL, b) can be denoted by logit(p) = -2.8 + 0.04 x a − 0.01 x b, with a cutoff value of logit(p) ≥ -2.3 and a ROC area of 0.82 ((95% CI 0.79 to 0.84, sensitivity = 76%, specificity = 73%).

A receiver operating characteristic (ROC) curve analysis was used to calculate the optimal predictor’s cut-off combination. Using the single predictor voided volume ≤ 220 mL derived from uroflowmetry, we can obtain a ROC area of 0.80  (Fig. 2a). Combined the predicted logit transformation of the probability of BOO, logit(p), for a voided volume (mL, a) and the presence of symptoms of VD (0 or 1, b) can be denoted by logit(p) = -1.3–1.1 x a + 0.014 x b, with a cut-off value of logit(p) ≥ -2.9 and a ROC area of 0.83 (Fig. 2b) (sensitivity = 75%, specificity = 77%).

For the factors affecting DU, multivariate logistic regression analysis found that age, MUCP, FPL, voided volume, and PVR were independent predictors for DU (Table 3). Nevertheless, the clinical symptoms of VD were not. A ROC curve analysis showed the following optimum cut-off value: (1) age ≥ 60 years old, which had a ROC area of 0.70 (Fig. 2c). (2) MUCP ≤ 50 cmH2O, which had a ROC area of 0.64 (Fig. 2d). (3) FPL ≤ 2.5 cmH2O, which had a ROC area of 0.61 (Fig. 2e). (4) Voided volume ≤ 200 mL, which had a ROC area of 0.79 (Fig. 2f). (5) PVR ≥ 30 mL, which had a ROC area of 0.63 (Fig. 2 g). To build a usable clinical model, the logit transformation of the probability of DU, logit(p), for age (year-old, a) and voided volume (mL, b) can be denoted by logit(p) = -2.8 + 0.04 x a − 0.01 x b, with a cutoff value of logit(p) ≥ -2.3 and a ROC area of 0.82 (Fig. 2h). Thus, although a urodynamic study may still be warranted to make a definite diagnosis, women who fulfill these criteria can be screened in an outpatient department.

**Table 3:** Prediction of detrusor underactivity in women with lower urinary tract symptoms (*n* = 1,886).

Variables	Univariate analysis		Multivariable analysis	
	Odds ratio (95% CI)	P^a^	Odds ratio (95% CI)	P^b^
Age (years)	1.061 (1.047–1.075)	< 0.001	1.037 (1.022–1.052)	< 0.001
Parity	1.285 (1.179–1.401)	< 0.001	–	–
Pad weight (g)	1.007 (1.003–1.011)	< 0.001	–	–
Strong desire (mL)	0.567 (0.464–0.795)	< 0.001	–	–
MUCP (cmH_2_O)	0.985 (0.980–0.991)	< 0.001	0.992 (0.986–0.997)	0.004
FPL (cm)	0.658 (0.559–0.788)	< 0.001	0.799 (0.663–0.963)	0.018
PTR (%)	1.000 (0.997–1.004)	0.680	–	–
Voided volume (mL)	0.338 (0.280–0.408)	< 0.001	0.388 (0.320–0.471)	< 0.001
PVR (mL)	1.531 (1.266–1.853)	< 0.001	1.565 (1.255–1.951)	< 0.001
Symptoms of VD	1.388 (0.942–2.045)	0.097	–	–

Values are expressed by number (percentage).

CI = confidence interval; FPL = functional profile length; MUCP = maximum urethral closure pressure; PTR = pressure transmission ratio; PVR = post-void residual volume; VD = voiding dysfunction.

^a^Univariate logistic regression analysis.

^b^Multivariable Stepwise backward logistic regression analysis was performed using those variables at univariate analysis. Pseudo R^2^ = 0.21.

## Discussion

Our study is one of the larger cohorts presenting the uniquely age-stratified prevalence of LUTS women who underwent UDS to evaluate DU and BOO. The total prevalence was 10.0% (189/1,886) of DU regardless of their clinical VD symptoms, and the total prevalence was 4.1% (77/1,886) of BOO in LUTS women. In addition, we developed a model calculated by voided volume and clinical VD symptoms to predict female BOO and DU. In this study, positive clinical VD symptoms were defined as any one or more of the positive descriptions of weak urinary stream, intermittency, strain to urination, and sensation of not emptying. Compared with the ICS definition: “abnormally slow and/or incomplete micturition”^8^, our positive clinical VD symptoms are slightly broader and clearer. The simple calculation can be implemented in clinical practice and aids doctors in diagnosing and prescribing first-line medication in the office setting.

The DU group showed an increasing trend along with age, while the BOO group kept a similar proportion throughout all age stratifications. Our cohort’s prevalence was 4.1% (77/1,886) of BOO in LUTS women. Farrar et al. reported a 6.7% prevalence of diagnosed BOO based on a sole peak flow rate of < 15 mL/s^9^. Blaivas and Groutz created a nomogram to diagnose BOO and classified its severity based on pressure-flow criteria of a free Qmax ≤ 12 mL/s and a Pdet. Qmax ≥ 20 cmH2O was associated with radiographic evidence of obstruction and reported a prevalence of 8.3%^10^. Gammie et al. reported a 9.2% BOO prevalence rate in the cross-section study. They used the same urodynamic criteria and excluded clinical obstruction for the DU and Normal groups women^7^. In our study, after excluding cystocele, we chose relatively strict criteria to avoid overlap but simple for ease of implementation.

Although there are different criteria to define DU, Jeong et al. compared several DU criteria, and the prevalence of the criteria we chose was 6.4%^7,11,12^. The result is consistent with another study as well. D’Alessandro et al. reported a prevalence between 3.7%~37% in 2,092 LUTS women attending the urogynecology clinic^13^. Few studies in the literature reported a detailed age stratification today. We showed that the age-stratified prevalence rate of DU increased from 4.2% at age 41–50 to 12.6% at age 61–70 and 47.5% at age over 70. In Osman’s review, the DU prevalence associated with age ranges from 9% (18–45 years old) to 48% (> 70 years old)^14,15^.

Our study found that the clinical VD symptoms can predict the diagnosis of BOO. Previous literature showed that storage symptoms like urinary frequency are the most frequent in BOO patients^16^. Indeed, 88% of BOO and 87% of DU patients in our cohort had storage symptoms such as frequency, urgency, or nocturia (data not shown). The high prevalence of bladder storage symptoms obscures its utilization to differentiate DU from BOO. The clinical VD symptoms in our cohort are more appropriate to predict BOO (*p* < 0.0001) instead of DU (*p* = 0.097). Using solely clinical VD symptoms as predictors of BOO has an odds ratio of 3.64 in this study (Table 1). Combined with a low voided volume (≤ 220 mL) by non-invasive uroflowmetry, the prediction of BOO can achieve a 0.83 ROC area.

The clinical VD symptoms cannot predict DU in our study. Our clinical DU prediction model used the age and voided volume by uroflowmetry. Uroflowmetry is a non-invasive measure of voiding pattern and amount. The logit transformation prediction model predicts a 0.82 ROC area. Previous literature used post-void volume (ROC = 0.78) or Qmax (ROC = 0.74) by a pressure-flow study^17^. The flow rate curve shape was investigated to approach DU and BOO patients. But no patterns can predict female DU, while prolonged and plateau way positively indicates female BOO^18^. In men with LUTS, a prediction model of DU used older age, smaller prostate volume, two symptoms (less urgency and a weak stream), and a lower maximum flow rate^19^. Compared with those prediction models, our prediction model is more straightforward, more accessible, and has a similar predictive ability.

Our study showed that MUCP is slightly higher in BOO and lower in DU. These results indicate a potential diagnostic ability to differentiate these two disease entities. The diagnostic value of MUCP had been shown in some types of BOO like high tone non-relaxing urethral sphincter^3,20^. The DU, on the contrary, was caused by the low detrusor contractility and had never been shown to correlate with the MUCP. Whether it is a physiologic change to the low contractility or secondary to aging is not known now. The role of MUCP in DU requires further research. In our study, we do not intend to incorporate the urethral pressure profilometry into our model because it is invasive and difficult to evaluate in the office-based situation.

The study’s strength is that this is a large cohort with 1,886 women. A sophisticated doctor uses easy-to-remember acronyms to ask about the clinical VD symptoms^21^, which ensures high inquiry consistency in this study. The limitation of our study is that only women with LUTS visited the hospital. We are unaware of the DU or BOO prevalence in asymptomatic healthy subjects. Second, the DU diagnosis criteria we selected might not be suitable to compare with other studies selecting different criteria. Third, the single voided volume of uroflowmetry may have relatively lower reproducibility. Repeated uroflowmetry or voiding diary is suggested to improve the limit.

Recalling the age-stratified prevalence of our study, the clinicians should be alert to the fact that elderly or menopausal women may have DU or BOO without clinical VD symptoms. A non-invasive uroflowmetry with voided volume can help predict the BOO. A higher percentage of BOO was found in women with the symptoms of VD. BOO can be predicted with low voided volume (less than 220 mL) and the presence of symptoms of VD. The DU could be predicted with the age and voided volume. Other suspicions warrant needing a urodynamic study or referral to a urogynecologic specialty.

## Methods

Between February 2005 and December 2020, the medical records of all women with LUTS but without cystocele who visited the urogynecology department of a medical center were reviewed. Our UDS inclusion criteria were LUTS symptoms. The exclusion criteria were cystocele, neurogenic bladder, previous anti-incontinence surgery, bladder tumor or stone, previous cervical cancer surgery, dementia, Parkinson’s disease, and psychotic patients. This study received approval from the National Taiwan University Hospital’s research ethics committee (REC No:202105082RINA). All experiments were performed in accordance with relevant guidelines and regulations. Additionally, this study was registered at ClinicalTrials.gov (NCT04981080).

A total of 1,886 women were included in this study. Informed consent for the examinations was obtained from all the women. Their baseline demographic data had age, parity, and history of overactive bladder syndrome (OAB) and stress urinary incontinence (SUI). OAB was defined as urinary urgency, with or without urgency incontinence, usually accompanied by increased urinary frequency and nocturia for at least three months^8^. All participants completed a 3-day bladder diary for subjective symptom records, such as daytime frequency, nocturia, urgency, and incontinence episodes^22^. The urodynamic study (UDS) was performed according to the recommendations of the International Continence Society^23^. The UDS includes non-instrumented uroflowmetry, filling cystometry was performed with 35 °C distilled water at a rate of 60 mL/sec, a pressure-flow study, and stress urethral pressure profilometry, was performed by an experienced technician with the patient in a sitting position according to the recommendations of the International Continence Society. Multichannel water-filled urodynamic equipment (Life-Tech, Houston, TX, USA) with computer analysis and Urovision (Urolab Janus System V, Houston) was used. Those women who have no complete data on the maximum flow rate (Qmax), voided volume, post-void residual volume (PVR), and detrusor pressure at a maximum flow rate (Pdet.Qmax) were excluded from this study.

DU was defined as when the detrusor pressure at the maximum flow rate (Pdet.Qmax) was < 20 cmH_2_O, the maximum flow rate (Qmax) was < 15 mL/s, and the bladder voiding efficiency (BVE, voided volume/ (voided volume + postvoid residual volume)) was less than 90% ^7^. BOO was defined when the detrusor pressure at the maximum flow rate (Pdet.Qmax) was ≥ 40 cmH_2_O and the maximum flow rate (Qmax) was < 12 mL/s^7^. Women who met the above diagnostic criteria were allocated to the DU or BOO group. Those women without DU or BOO were distributed to the non-DU and non-BOO groups.

The presence of clinical symptoms defines the VD symptoms. Formulated questions were asked in the outpatient department. The formulated questions are in two main categories: OAB and VD. Frequency, urgency, and nocturia (acronym FUN) were asked to evaluate the bladder storage symptoms. Weak urinary stream, intermittency, strain to urination, and sensation of not emptying (acronym WISE) of the bladder symptoms were used to define VD according to the FUN-WISE acronym^21^. Women with any one or more of the positive descriptions of WISE were recorded as with clinical symptoms of VD.

The primary outcome of this study was the correlation of DU, BOO, non-DU, and non-BOO in women with or without clinical symptoms of voiding dysfunction. Age-specific prevalence in women with and without VD was collected and analyzed. The secondary outcomes were the prediction factors among these four groups in subjective VD symptoms and objective UDS parameters. A prediction model was built.

STATA software (Stata Corp., College Station, TX, USA) was used for statistical analysis. One-way ANOVA was performed using GraphPad Prism version 10.0.0 for MacOS (GraphPad Software, Boston, Massachusetts USA, www.graphpad.com). The chi-square, univariate, and multivariable logistic regression tests were used for statistical analysis as appropriate. A *p-value* of less than 0.05 was considered statistically significant.

## Data Availability

The datasets analyzed during this study are available from the corresponding author upon reasonable request.

## Abbreviations

BOO: Bladder outlet obstruction
DU: Detrusor underactivity
ICS: International Continence Society
LUTS: Lower urinary tract symptoms
MUCP: Maximum urethral closure pressure
OAB: Overactive bladder syndrome
Pdet.Qmax: Detrusor pressure at a maximal flow rate
POP: Pelvic organ prolapse
POP-Q: Pelvic organ prolapse quantification
PVR: Post-void residual urine
Qmax: Maximal flow rate
ROC curve: Receiver operating characteristic curve
SUI: Stress urinary incontinence
UDS: Urodynamic study
VD: Voiding dysfunction
